# Bioactive Compounds and Antioxidant Potential of Truffles: A Comprehensive Review

**DOI:** 10.3390/antiox14111341

**Published:** 2025-11-07

**Authors:** Sara Baldelli, Gilda Aiello, Alessandra De Bruno, Serena Castelli, Mauro Lombardo, Vilberto Stocchi, Gianluca Tripodi

**Affiliations:** 1 Department for the Promotion of Human Science and Quality of Life, San Raffaele Open University, Via di Val Cannuta, 247, 00166 Rome, Italy; alessandra.debruno@uniroma5.it (A.D.B.); mauro.lombardo@uniroma5.it (M.L.); gianluca.tripodi@uniroma5.it (G.T.); 2 IRCCS San Raffaele Roma, 00166 Rome, Italy

**Keywords:** truffles, bioactive compounds, antioxidant activity, anticancer activity, nutritional value, extraction techniques

## Abstract

Truffles are edible symbiotic hypogeal fungi and highly prized worldwide for their unique aroma and rich nutritional profile. Belonging to the order Pezizales and family Tuberaceae, with the genus Tuber being the most notable, truffles contain a diverse array of bioactive compounds including phenols, terpenoids, polysaccharides, anandamide, fatty acids, and ergosterols. These compounds contribute to a wide range of biological activities such as antioxidant, antibacterial, anti-inflammatory, hepatoprotective, and anticancer effects. This review comprehensively summarizes current scientific evidence on the biochemical composition, nutritional and aromatic properties, and biological activities of truffles, with special emphasis on their antioxidant and anti-tumor potential. Additionally, factors influencing truffle productivity and quality as well as advanced extraction and storage techniques to preserve bioactivity are discussed, highlighting their potential as valuable functional foods and sources of natural antioxidants.

## 1. Introduction

Truffles, the underground fruiting bodies of ectomycorrhizal fungi, represent a unique convergence of culinary luxury and biochemical complexity. Celebrated worldwide for their intense aroma and rarity, species of the Tuber genus fetch some of the highest prices per kilo in the gourmet food market [1]. This extraordinary value is not only gastronomic, but also economic and ecological, supporting rural economies and symbiotic forest ecosystems in Europe, North Africa, Asia, and parts of North America. The global truffle market, driven by species such as *Tuber melanosporum* (Périgord black truffle) and *Tuber magnatum* (Italian white truffle), has fueled growing interest in truffle cultivation, chemical ecology, and biotechnological exploitation.

Truffles belong to the phylum Ascomycota, order Pezizales, and family Tuberaceae, with the Tuber genus being the most economically significant. These fungi form ectomycorrhizal relationships with host trees such as oaks, hazelnuts, and poplars, a relationship crucial not only for the development of the fruiting body but also for its secondary metabolic profile. Despite being taxonomically diverse, truffles display convergent biochemical traits, including a rich profile of volatile organic compounds, a high phenolic content, and a recently discovered endocannabinoid system. Compositionally, truffles are distinguished by a rich and varied profile: complex carbohydrates, high biological value proteins, unsaturated fatty acids, sterols, water- and fat-soluble vitamins, as well as essential minerals [2]. These compounds are complemented by phenols, flavonoids, terpenes, polysaccharides, and volatile compounds, which are responsible not only for their distinctive aroma but also for significant biological activities. Numerous experimental studies have demonstrated that these molecules possess antioxidant, anti-inflammatory, antimicrobial, antiviral, and hepatoprotective properties. Furthermore, more recent studies have begun to highlight the anticancer and immunomodulatory potential of specific extracts or polysaccharide fractions [3]. At the same time, technological research has developed innovative extraction and preservation methods (such as ultrasound-assisted extraction, supercritical fluids, and encapsulation) aimed at preserving the stability of bioactive compounds and expanding their potential industrial applications [4]. This has opened up prospects not only in the food sector, but also in the nutraceutical and pharmaceutical fields, making truffles a multifunctional resource of great scientific and economic interest. In light of this evidence, this review aims to provide an updated overview of the nutritional and chemical characteristics of truffles, with particular attention to their main bioactive compounds, documented biological activities, and the most recent extraction and preservation strategies. The goal is to highlight the potential of these fungi as a natural resource for the development of new functional and therapeutic products, as well as a valuable food in the culinary tradition.

### Literature Search Strategy

The literature included in this review was selected through a comprehensive search of the PubMed database. The search strategy combined the following keywords and their Boolean variations: “truffle”, “bioactive compounds”, “antioxidant activity”, “anticancer and antioxidant activities”, “nutritional values”, and “extraction technique”. The search covered publications from January 2000 to July 2024. Only peer-reviewed articles written in English were considered. Studies focusing on truffles from the genera *Tuber*, *Terfezia*, *Tirmania*, and *Picoa* were included. Exclusion criteria were (i) conference abstracts without full text, (ii) non-peer-reviewed materials, (iii) studies not directly investigating the biochemical composition or biological activity of truffles, and (iv) duplicate records. The selection process prioritized the most recent and methodologically robust studies to ensure an updated and reliable overview of truffle bioactivity and composition.

## 2. Chemical Composition of Truffles

On a dry weight basis (DW), truffles are predominantly composed of carbohydrates (ranging from 37% to 80%), followed by proteins (11% to 35%), with lower levels of fats (2% to 7%) and fibers (4% to 13%) [3,5,6,7]. They are also rich in essential minerals (notably potassium, phosphorus, calcium, magnesium and iron) [8].

The mineral content can vary by species and region, but potassium, phosphorus, and calcium are the predominant macroelements, accounting for the majority (≈80–90%) of total mineral content [9]. Truffles contain a favorable lipid profile dominated by unsaturated fatty acids, which are associated with cardiovascular and anti-inflammatory health benefits [2]. Truffle lipids exhibit a favorable fatty-acid profile, with monounsaturated and polyunsaturated fatty acids (MUFA and PUFA) often exceeding 60% of total fatty acids. Alongside their lipid content, truffles are rich in bioactive compounds, including phenolics (e.g., catechin, epicatechin), flavonoids (e.g., kaempferol, baicalein), and terpenoids, which provide potent antioxidant, antimicrobial, and anti-inflammatory activities [10]. The distinctive aroma of truffles is attributed to a complex mixture of volatile organic compounds (VOCs), especially sulfur-containing molecules. Truffles present a complex mixture of bioactive and nutritional compounds, whose profiles vary by species, maturation stage, geographic origin, and extraction method. To provide a concise comparative overview of the macronutrient profile and key bioactive constituents among selected truffle species, Table 1 provides a comparative overview of the nutritional composition and key bioactive compounds found in various truffle species. Moreover, the macronutrient and mineral profile of truffles is both species- and region-dependent, as demonstrated by *Tuber indicum*. Samples collected from different regions of China show notable compositional differences: those from Jilin contain the highest carbohydrate levels (≈52%), those from Sichuan have the highest protein content (≈23%) and ergosterol levels, while samples from Yunnan exhibit the highest fat content (≈6.6%) [11].

### 2.1. Polysaccharides and Bioactive Fibers

Truffle polysaccharides represent a major fraction of their dry matter and exhibit remarkable structural diversity. They include β-glucans, homoglucans, heteropolysaccharides, chitin, and other non-starch polymers [17]. Their monosaccharide composition is dominated by glucose, mannose, rhamnose, and galactose, with glucose often accounting for more than 90% of the total content in some species [18]. Both neutral and acidic fractions have been reported, with molecular weights ranging from a few kDa up to more than 700 kDa, depending on the truffle species and the extraction protocol used. In *Tuber panzhihuanense* and *Tuber pseudoexcavatum*, polysaccharide fractionation yielded a neutral polysaccharide (NP) of approximately ~5.1 kDa, mainly composed of mannose and rhamnose, and two acidic fractions (I and II) with molecular weights of ~30.8 kDa containing mannose, rhamnose, galactose, and glucose [18]. In contrast, *Tuber sinense* yielded a highly pure, water-soluble homopolysaccharide (PTS-A) with a molecular weight of ~728 kDa, consisting exclusively of α-D-glucose residues linked via (1→6) glycosidic bonds, highlighting the structural variability within the genus *Tuber* [18]. Collectively, these results reveal that truffle polysaccharides span a wide spectrum of molecular architectures and linkage patterns. Glucose-rich β-glucans and α-glucans form the primary structural backbone, while mannose-, rhamnose-, and galactose-containing heteropolysaccharides contribute to matrix complexity and potential prebiotic functionality. Such polymers represent not only structural components but also bioactive dietary fibers that modulate physiological responses. Another study on *Tuber sinense* PTS-A has demonstrated strong activity in DPPH and superoxide radical scavenging assays with IC_50_ values in the low mg/mL range [18]. The bioactivity of truffle polysaccharides appears to be closely linked to molecular weight, branching degree, monosaccharide composition, and glycosidic linkage type, particularly the presence of α-(1→6) linkages, which are often associated with enhanced radical-scavenging and immunomodulatory effects. These findings highlight the dual nutritional and functional role of truffle polysaccharides, bridging structural fiber components and bioactive macromolecules relevant to human health.

### 2.2. Lipids: Fatty Acids and Ergosterols

Truffles are relatively low in lipids compared to other macronutrients, yet their lipid fractions are chemically diverse and biologically relevant. The total fat content of truffles varies between species and geographic origin, typically ranging from 1.8% to 7.4% on dry weight basis [5]. The lipid composition varies based on species, region, maturity stage, and post-harvest treatment, making it a promising chemotaxonomic and nutritional marker. Truffles contain a diverse range of fatty acids, with unsaturated fatty acids (notably oleic and linoleic acids) often accounting for more than 60% of total fatty acids in many species [3,19]. The ratio of saturated to unsaturated fatty acids varies by species, but polyunsaturated fatty acids are frequently dominant [2]. Species such as *Terfezia claveryi*, *Terfezia boudieri*, *Tuber melanosporum*, and *Tirmania nivea* are particularly rich in oleic and linoleic acids, whereas *Tuber aestivum* also contains notable levels of palmitic (C16:0) and elaidic (C18:1 trans) acids [20]. *Tuber magnatum*, in contrast, tends to exhibit a higher proportion of saturated fatty acids compared to other *Tuber* species [10]. In general, monounsaturated fatty acids (MUFAs) like oleic acid represent 30–60% of the total lipid fraction, polyunsaturated fatty acids (PUFAs) such as linoleic acid make up 20–40%, while saturated fatty acids (SFAs), including palmitic and stearic acids, account for 10–20% [3]. Advanced lipidomic profiling using UHPLC-QE Orbitrap-MS has identified over 30 lipid molecular species, including lysophosphatidylcholines (LPCs), triacylglycerols (TGs), phosphatidic acids (PAs), and cardiolipins (CLs). These not only contribute to membrane structure and signaling but may also interact with volatile organic compounds (VOCs), influencing truffle aroma [21]. Truffles also contain sterols such as ergosterol, brassicasterol, and stigmasterol, which can serve as taxonomic markers [22]. Sterol analysis has revealed the consistent presence of ergosterol, the fungal sterol analog of cholesterol, as well as β-sitosterol and brassicasterol. These compounds are not only markers of fungal identity but also possess antioxidant and cholesterol-lowering properties [22,23]. Notably, ergosterol levels in some *Tuber* spp. range from ~1.28 to 1.80 mg/g dried matter [22]. Ergosterol is not only a structural component of fungal membranes but also a precursor of vitamin D_2_ upon UV exposure in some fungi; its presence may have nutritional and bioactive implications. Variation among species is notable: *Tuber aestivum* shows higher ergosteryl ester content than *Tuber melanosporum* and *Tuber indicum* [22]. Also, some lipid oxidation products likely feed into volatile compound pathways, contributing to aroma as lipids degrade during maturation or storage.

### 2.3. Phenolic Compounds

Truffles are rich in phenolic compounds, which contribute to their antioxidant, anti-inflammatory, and potential health-promoting properties [10]. The total phenolic content in various truffle species typically ranges from about 1 to 43 mg per gram of dried matter, depending on the species and extraction method [22,24]. For example, *Tuber magnatum* exhibits TPC of ~290 mg gallic acid equivalents (GAE) per 100 g fresh weight in certain populations, far higher than many other Tuber species [2]. In a comparative study of *Tuber melanosporum*, *Tuber aestivum*, and *Tuber indicum*, TPC (Folin–Ciocalteu assay) fell in the range of approximately 1–2 mg GAE per g dried matter [22]. Identified phenolic acids and derivatives in truffles include gallic acid, homogentisic acid, protocatechuic acid, p-hydroxybenzoic acid, o- and p-coumaric acids, syringic acid, trans-cinnamic acid, and flavonoids such as baicalein, kaempferol, epicatechin, catechin, and myricetin [10,22,24]. Quantification of these indicates that hydroxycinnamic acid derivatives (e.g., coumaric acids) are present in *Tuber aestivum* but either absent or negligible in *Tuber melanosporum* and *Tuber indicum* [22]. Functional assays correlate phenolic content with inhibition of lipid oxidation (linoleic acid systems) specifically, *Tuber aestivum* extracts showed ~24% inhibition, *Tuber indicum* ~23%, *Tuber melanosporum* ~18% [22].

### 2.4. Vitamins

Truffles are a valuable source of essential vitamins, with species-specific differences that contribute to their nutritional and functional properties. Vitamin C is particularly abundant in desert truffles such as *Terfezia claveryi* and *Tirmania nivea*, where it has been quantified up to 5.1% of dry weight, providing strong antioxidant and immune-protective effects [5]. By contrast, European species such as *Tuber melanosporum* and *Tuber magnatum* generally exhibit lower concentrations of vitamin C, but still contribute significantly to the antioxidant potential of their fruiting bodies [2]. Truffles also contain B-complex vitamins, including thiamine (B1), riboflavin (B2), niacin (B3), and folate (B9), although their levels vary across species, i.e., *Tuber aestivum* and *Tuber magnatum* are reported to be particularly rich in niacin and riboflavin, whereas desert truffles such as *Terfezia boudieri* and *Tirmania nivea* show higher levels of thiamine and folate [12]. Vitamin E is present in *Terfezia* species, and β-carotene (a precursor of vitamin A) is found in both *Terfezia* and *Picoa* species [25]. Alongside these vitamins, truffles provide additional antioxidant molecules, including anthocyanins and phenolic compounds, which further enhance their health-promoting potential. Of particular interest are ergosteroids, which can be metabolized into vitamin D in humans, adding another dimension to the nutraceutical relevance of truffles [26]. Together, these findings indicate that desert truffles are generally richer in vitamin C and folates, while European black and white truffles contribute predominantly B vitamins, β-carotene, and vitamin D precursors, highlighting their dual value both as gourmet delicacies and as functional dietary resources.

### 2.5. Mineral Content

The mineral profile is dominated by potassium, followed by phosphorus and magnesium; iron and zinc are present in smaller quantities and vary depending on the terroir [9]. The bioavailability of minerals in truffles appears to be mainly influenced by the chitin-glucan matrix present in the cell wall, rather than by phenolic chelators. Furthermore, co-consumption with organic acids or animal proteins can improve the intestinal absorption of these minerals [27]. Adding a serving of mushrooms to the diet can significantly increase potassium intake (by 12–14%) without affecting sodium levels, further improving the K/Na ratio and supporting cardiovascular health [28].

### 2.6. Endocannabinoid Compounds (e.g., Anandamide)

The discovery of anandamide and system components of endocannabinoid metabolism in *Tuber melanosporum* is among the most novel findings. In *Tuber melanosporum*, at maturation stage VI, transcripts and proteins for key metabolic enzymes such as NAPE-PLD (synthetic) and FAAH (degradative) are expressed, though canonical cannabinoid receptors (CB1, CB2) are not measurably expressed [29]. LC-MS quantification across maturation stages showed increasing anandamide content with advancing maturity. The other major endocannabinoid 2-arachidonoyl glycerol (2-AG) was typically below the detection limit [29]. The presence of anandamide in truffles is thought to be evolutionarily ancient and may serve as an attractant for animals, including humans, who possess the necessary receptors to experience its effects. Hypotheses for functional role include that anandamide acts as an olfactory attractant for mammals that aid in spore dispersal, leveraging mammal endocannabinoid receptors. Also, given anandamide’s known bioactivities (analgesic, modulatory of mood, reward pathways), its dietary presence may have physiological implications yet to be thoroughly tested [29]. While the content of endocannabinoids like anandamide in truffles is notable, no evidence consuming truffles produces cannabis-like effects in humans, as the concentrations are likely too low and the necessary receptors are not present in the fungi themselves [29,30]. Overall, the discovery of anandamide in truffles highlights a unique intersection between fungal biochemistry and the mammalian endocannabinoid system [3,29].

## 3. Nutritional Value and Organoleptic Properties

### 3.1. Protein and Amino Acid Profile

Truffles provide a moderate protein intake at fresh weight but a high-quality amino acid spectrum on a dry basis, with enrichment in glutamate/aspartate and sulfur-containing amino acids that act as precursors of both umami and the main sulfides/aromas [3]. The protein content ranges from about 16% to 27% of dry weight depending on species, origin, and maturity [5,31]. For example, *Terfezia claveryi* and *Tirmania nivea* contain 19.6% and 27.2% protein, respectively, while *Terfezia arenaria* has about 14% protein [5,31,32]. Truffle proteins are of high quality, containing all essential amino acids in appreciable amounts; in *Terfezia claveryi*, leucine and lysine are the first limiting amino acids, while in *Tirmania nivea*, valine is limiting [5]. In *Terfezia boudieri*, 29 amino acids have been detected, and the essential amino acids represent about 6% of the dry weight [31,33]. Comparative analyses across genera reveal marked species-level differences. Species-level differences are well documented: *Tuber japonicum* is rich in glutamine and glutamic acid, while *Tuber magnatum* contains a large amount of alanine, and *Tuber melanosporum* generally has higher overall amino acid yields compared to other truffles [34,35]. The maturation stage plays a significant role in protein and amino acid composition: mature ascocarps show a broader amino acid spectrum and a more complex proteomic profile, consistent with increased metabolic and enzymatic activity during ripening [36,37].

From a nutritional standpoint, truffle proteins are highly digestible. In vitro digestion assays indicate digestibility rate between 82.8% and 86.7%, and the protein efficiency ratio is close to that of casein, a high-quality animal protein [5]. Simulated gastrointestinal digestion studies suggest that chitin and β-glucans present in the fungal cell wall partially limit proteolysis by reducing enzyme accessibility; however, mild thermal processing, enzymatic pre-treatment, or micronization significantly enhance peptide release and amino acid bioaccessibility without altering the volatile or sensory profile [38]. In addition to their nutritional contribution, truffle-derived peptides and free amino acids play a key role in sensory perception. Free glutamate, aspartate, and sulfur-containing amino acids (e.g., methionine, cysteine) act as biochemical precursors for volatile sulfur compounds such as bis(methylthio)methane, which defines the characteristic truffle aroma [39]. Moreover, 5′-ribonucleotides such as inosine monophosphate (IMP) and guanosine monophosphate (GMP) interact synergistically with glutamate, enhancing the umami intensity even at sub-millimolar concentrations [39].

Recent proteomic and peptidomic investigations have also identified bioactive peptides in *Tuber* and *Terfezia* extracts with potential antioxidant, antihypertensive, and immunomodulatory properties, further expanding the nutritional relevance of truffle proteins. These peptides may contribute to truffles’ reputation as functional gourmet foods that combine sensory complexity with biochemical benefits. Research has demonstrated that protein hydrolysates from *Terfezia claveryi* exhibit strong antioxidant and antimicrobial activities, with some peptide fractions outperforming the original proteins in reducing oxidative stress and inhibiting microbial growth [40]. Overall, truffles provide a complete and digestible protein source, with a diverse amino acid profile that supports both nutritional value and their distinctive sensory properties [5,35,37].

### 3.2. Aromatic Characteristics and Impact on Gastronomic Value

Truffle aroma is one of their most distinctive features, underpinned by more than 200 volatile organic compounds (VOCs) across species, though within a single species, only ~30–60 are aroma-active. Sensory identity in truffles is defined by a subset of odor-active volatiles with odor activity values (OAV) > 1, primarily sulfur compounds (e.g., methanethiol, disulfides, DMS), 1-octen-3-one/-ol, short-chain aldehydes and ketones; less abundant thiophenes, pyrazines, phenols, and terpenoid fragments contribute to species-specific nuances [41,42]. Some VOCs are shared among many species (e.g., DMS, 1-octen-3-ol), others are species-specific. For example, white truffles like *Tuber magnatum* have volatiles described as more intense garlic, sulfuric, and honey-like, whereas *Tuber melanosporum* tends toward musky, earthy, and pungent aroma notes [42]. Gastronomically, typical portions (3–10 g) provide few calories but have a marked flavor-driven effect of reducing salt, through the glutamate–5′-nucleotide synergy and greater retronasal persistence in fatty matrices at 55–65 °C [43,44]. Prolonged or high-temperature cooking depletes sulfur volatiles such as thiols and disulfides, while enhancing oxidative aromatic notes; brief, low-temperature exposure is recommended to preserve the truffle’s aromatic integrity [45].

Based on recent findings using HS-SPME coupled with GC-MS and GC-Olfactometry (GC-O), several key odor-active compounds have been identified in *Tuber* species, with concentrations and odor activity values (OAVs) confirming their central role in truffle aroma. Among these, bis(methylthio)methane (BMTM) stands out as the dominant volatile in *Tuber magnatum*, accounting for up to 78% of the total headspace volatiles and displaying an extremely low odor detection threshold (<1 ppb), which gives it a high OAV and makes it a primary contributor to the characteristic garlic-like and sulfurous aroma of white truffles [46]. In contrast, 1-octen-3-ol, a common “mushroom-like” alcohol, is the principal odorant in *Tuber melanosporum* and *Tuber aestivum*, though with a higher odor threshold, requiring higher concentrations to be impactful [47]. Other significant volatiles include 3-methylbutanal and methional, which contribute malty and potato-like notes, respectively, especially in black truffles [47]. Ethylphenol and other phenolic compounds also impart leathery or smoky undertones, particularly in *Tuber melanosporum* [48]. Maturation and associated microbiota profoundly modulate the volatilome; metagenomic-chemical evidence indicates a bacterial contribution to key sulfides in the late stages [49].

The lipid matrix (unsaturated phospho/glycolipids) acts as a transient reservoir for sulfur VOCs, explaining the increase in intensity when truffles are conveyed in warm fats (egg, butter, milk) [50]. Moreover, post-harvest processes affect the aroma profile. Aromatic evolution occurs rapidly, as mass loss and enzymatic activity can modify VOCs within a few days, even at 0–2 °C [45]. Low O_2_ protective atmospheres slow down degradation but can “flatten” the aroma; short storage in micro-perforated paper at cold temperatures better preserves the top notes. Freezing whole alters the consistency and balance of VOCs; grating into fat and quick freezing partially stabilizes sulfides through lipid partitioning [51].

## 4. Biological Properties of Truffles

**Figure 1:**
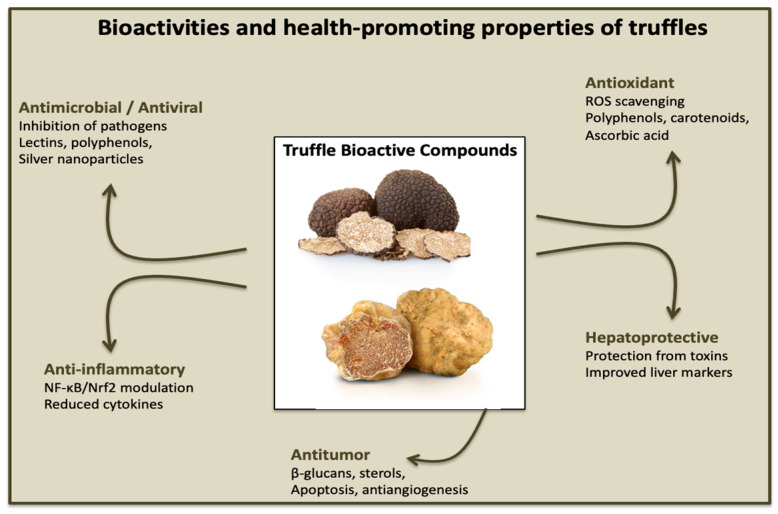
Bioactivities and health-promoting properties of truffles. Schematic representation of the main biological activities attributed to truffles and their bioactive compounds. Truffle-derived molecules, including polysaccharides, polyphenols, sterols, carotenoids, and terpenoids, exert multiple health-promoting effects. These comprise antimicrobial/antiviral activity, antioxidant and anti-inflammatory properties, hepatoprotective functions, and antitumor potential. Such pleiotropic effects highlight the relevance of truffles not only as culinary delicacies but also as promising sources of nutraceutical and therapeutic agents.

In addition to their culinary prestige, truffles also stand out for their numerous biological activities and health benefits that have attracted considerable scientific interest. In vitro and in vivo studies have confirmed the antioxidant, anti-inflammatory, antimicrobial, antiviral, hepatoprotective, and antimutagenic properties of these compounds. These characteristics not only enhance their nutritional value but also open up prospects for the development of value-added truffle-related products, eventually related to nutritional supplementation. The main bioactivities, supported by the scientific literature, are explored in this section and illustrated in Figure 1.

### 4.1. Antiviral, Antibacterial and Antimicrobial Effects

Among the various bioactivities attributed to truffles, those of antiviral and antimicrobial nature are among the most extensively investigated. Hussan and Al-Ruqaie have already highlighted that some species belonging to the genus Terfezia possess antiviral activities potentially relevant in the treatment of skin and eye diseases [52]. At the same time, numerous studies have documented the antimicrobial activity of desert truffles, particularly through agar diffusion tests. For example, aqueous extracts (5%) of *Terfezia claveryi* demonstrated a 40% inhibition of the growth of *Pseudomonas aeruginosa* [53], while concentrations between 4% and 10% of *Terfezia claveryi* and *Tuber nivea* showed an inhibitory activity ranging from 70% to 100% against *Pseudomonas aeruginosa* and *Staphylococcus aureus* [54]. Subsequent studies have also reported that *Terfezia boudieri* exhibits antimicrobial activity against both Gram-positive and Gram-negative bacteria, as well as against the pathogenic yeast *Candida albicans* [55]. Further evidence confirms the antimicrobial potential of other species: Janakat et al. found that the aqueous extract of *Terfezia claveryi* reduced the growth of *Staphylococcus aureus* by 66.4% [53]. Owaid et al. observed a marked antibacterial effect of silver nanoparticles synthesized by *Tirmania* sp. on Gram-positive and Gram-negative bacteria, with particular efficacy against *Pseudomonas aeruginosa* [56]; Casarica et al. demonstrated that the aqueous extract of *Terfezia claveryi* was active against *Escherichia coli*, *Staphylococcus epidermidis*, and *Staphylococcus aureus*, suggesting its potential therapeutic use in ocular infections [56]. In addition, *Tuber nivea*, widespread in the arid regions of Tunisia, showed a broad antimicrobial spectrum, active against three Gram-positive and four Gram-negative bacteria, including *Salmonella typhimurium*, *Escherichia coli*, *Pseudomonas aeruginosa*, *Enterococcus faecalis*, *Staphylococcus aureus*, *Staphylococcus epidermidis*, and *Bacillus subtilis* [57]. *Tuber pinoyi* has also shown significant activity: DibBellahouel and Fortas attributed the antimicrobial effect of its ethyl acetate extract to the presence of pyrazines and derivatives [58], while Stojković et al. documented that aqueous and methanolic extracts were able to limit the proliferation of *Staphylococcus aureus* in chicken broth stored both at room temperature and refrigerated [59] (Table 2).

Although the mechanisms responsible for the antimicrobial activity are not yet fully elucidated, the literature suggests the involvement of various fungal metabolites. In particular, lectins produced by truffles may be able to recognize and eliminate bacterial exopolysaccharides; fungal polysaccharides may help modulate microbial defense systems; while laccases would catalyze the oxidation of phenolic compounds, resulting in the release of superoxide radicals and the production of hydrogen peroxide, both potentially inhibitory for numerous pathogenic microorganisms [60,61,62]. As described in the section of this review concerning the chemical composition of truffles, these fungi contain high concentrations of polyphenols, anthocyanins, and carotenoids, which are well-known for their anti-inflammatory properties. Primarily, these compounds exhibit broad-spectrum antimicrobial activity, with a reduced likelihood of inducing resistance. Due to the advantages associated with the use of polyphenols in this context, antimicrobial phenolic biomaterials have been developed from natural polyphenols. These materials exploit not only the ability of polyphenols to reduce infection transmission, but also their capacity to remove bacterial biofilms and prevent the development of resistance, offering a promising therapeutic alternative in cases of antibiotic resistance [63]. This is a particularly relevant finding, considering that biofilm formation is one of the primary factors limiting antibiotic penetration and efficacy against bacterial populations. Furthermore, the use of truffle-derived compounds could be combined with conventional therapeutic approaches to enhance overall treatment efficacy [64]. Overall, the primary mechanism through which truffle-derived extracts exert their antimicrobial activity is linked to their antioxidant properties, making them useful for reducing the inflammatory process.

### 4.2. Antioxidant and Anti-Inflammatory Activities

As previously mentioned, several studies have highlighted that truffles represent a significant source of bioactive molecules, particularly phenolic compounds and polysaccharides, endowed with marked antioxidant activity [65]. Phenols are known for their ability to neutralize free radicals such as peroxyl radicals or singlet oxygen, thus helping to reduce oxidative damage [66]. Some polysaccharides isolated from species such as *T. indicum* have also shown a protective effect on PC12 cells subjected to oxidative stress induced by H_2_O_2_ [67]. Similarly, extracts of *Terfezia boudieri* showed effective scavenging activity against DPPH (2,2-diphenyl-1-picrylhydrazyl) radicals, with an IC_50_ value of 0.031 mg/mL [55], while polysaccharides from *Tuber huidongense* and methanolic extracts from *Tuber leonis* and *Tuber pinoyi* confirmed a high radical-scavenging capacity [68]. Among the main compounds responsible for these effects were catechin, ferulic acid, p-coumaric acid, and cinnamic acid [69].

In addition to this biochemical interest, the antioxidant profile of truffles is also particularly relevant from a nutritional perspective, as antioxidant-rich foods are associated with protection against oxidative stress mediated by reactive oxygen species (ROS). Indeed, among antioxidant compounds contained into truffles there are ascorbic acid, ergosterol, phenolics, flavonoids, terpenoids, phytosterol, and polysaccharides, whose antioxidant role has been extensively studied, particularly for its potential use as an adjuvant in anti-cancer therapies [3,70].

ROS increase is considered a key mechanism in the development of numerous degenerative diseases, including cardiovascular disease, cancer, inflammation, and aging. Some comparative studies have highlighted that fresh truffles possess a higher antioxidant content than other edible mushrooms. For example, *Terfezia claveryi* and *Picoa juniperi* showed higher activity in lipid peroxidation, deoxyribose, and peroxidase tests compared to common antioxidant additives such as α-tocopherol, BHA (butylated hydroxyanisole), BHT (butylated hydroxytoluene), and propyl gallate [71]. The ability to chelate iron may be of fundamental importance in reducing lipid peroxidation and the resulting ferroptosis. This aspect is particularly relevant for diseases characterized by elevated levels of ferroptosis, such as cancer, neurodegeneration, sepsis, ischemia–reperfusion injury, autoimmune disorders, and metabolic disorders [72]. However, the industrial processes such as freezing and canning result in a decrease in the antioxidant capacity of processed truffles, thus the processing methods applied to truffles prior to their direct consumption may play a crucial role in preserving both the beneficial properties of their bioactive compounds and their organoleptic characteristics [71].

Another interesting analysis conducted by Al-Laith [73] examined the antioxidant potential of desert truffles (*Tuber nivea*) collected from different geographical areas, including Bahrain, Iran, Morocco, and Saudi Arabia. Four different assays were used to evaluate antioxidant activity: FRAP (ferric reducing antioxidant power), DPPH (2,2-diphenyl-1-picrylhydrazyl free radical scavenging), deoxyribose assay, and nitric oxide (NO) radical inhibition. The results indicated that samples from Iran showed the highest DPPH radical scavenging capacity (30.6 ± 13%), the greatest efficacy against NO radicals (EC_50_ = 102 mg/mL), and the highest level of inhibition of deoxyribose degradation (91%). In contrast, truffles from Bahrain and Saudi Arabia achieved the highest FRAP scores, 18.62 and 18.06 mmol/100 g, respectively. This work, therefore, highlights the need for a thorough characterization of the specific truffle species used, as different species may exhibit distinct and varying degrees of biological activity. Additionally, the geographic origin of the truffle may play a crucial role in determining its chemical composition and, consequently, its functional properties.

The protective effect of white truffle mycelial products against hydrogen peroxide-induced oxidative stress was also evaluated in zebrafish embryos. High antioxidant activities were observed in both ethanol and water extracts (20 mg/mL) of mycelial products obtained after 3 weeks of solid-state fermentation of white truffle. In this regard, it has been demonstrated that the extraction method significantly influences the antioxidant capacity of truffle-derived extracts. Specifically, aqueous extracts obtained from the same truffle species exhibit higher antioxidant activity compared to ethanol extracts [74].

Several bioactive compounds present in truffles, including phenols, terpenoids, and polysaccharides, not only exert antioxidant effects but are also able to modulate inflammatory processes [75]. Oxidative stress is known to be closely linked to chronic inflammatory conditions, such as diabetes mellitus (DM), in which hyperglycemia leads to excessive production of free radicals [76]. The relationship between oxidative stress and inflammation is bidirectional, as inflammation itself can also lead to an increase in intracellular ROS levels. In certain cases, such as those involving myeloperoxidase and NADPH oxidases, this ROS production is part of a proper immune response, with immune cells utilizing ROS to induce the death of exogenous bacteria and viruses [77]. In the context of metabolic disorders, Zhang et al. observed that the administration of an aqueous extract of *Tuber melanosporum* to rats made hyperglycemic by streptozotocin reduced blood glucose levels, with an efficacy comparable to that of the reference drug glibenclamide. The hypoglycemic action of truffle was associated with the activation of the Nrf2 and NF-κB pathways and the enhancement of antioxidant defenses, both enzymatic (superoxide dismutase, catalase) and non-enzymatic (vitamins C and E) [78]. Interestingly, a neuroprotective role of the black winter truffle has been highlighted in diabetic rats. According to the literature, diabetic rats exhibit higher levels of α-synuclein, particularly in its monomeric and misfolded forms, which are responsible for the formation of toxic oligomers. These oligomers, in turn, have been shown to play a significant role in neurodegenerative diseases, more specifically in Parkinson’s disease, and are associated with dysregulation of the insulin signaling pathway and oxidative stress. Tuber brumale, or black winter truffle, acts by reducing α-synuclein accumulation, mainly due to its antioxidant properties [79]. A similar role has been observed for Tuber sinense, a black truffle that has demonstrated the ability to act as a free radical scavenger in neuronal cells. This activity has beneficial effects on sleep quality in Drosophila; in fact, the total sleep time is prolonged following the use of Tuber sinense extracts, and sleep is also promoted in insomnia-like conditions [80]. This ability is especially important considering that oxidative stress plays a crucial role in the development and progression of neurodegenerative disorders, also impacting the health of associated muscle tissue [77]. This suggests that the use of truffle extracts and their derived peptides holds significant potential in the context of neurodegenerative diseases and dementias. For example, in scopolamine-induced dementia, truffles have shown beneficial effects on memory performance [81]. The ability of truffle extracts and their peptides to cross the blood–brain barrier makes them even more promising from a therapeutic perspective [80,81]. In addition to this metabolic effect, Janakat and Nassar reported the hepatoprotective activity of the aqueous extract of *Terfezia claveryi* against oxidative damage in the liver [82]. Further evidence was provided by Beara et al. [10], who demonstrated that *Tuber magnatum* extract can inhibit pro-inflammatory metabolites derived from the COX-1 and 12-LOX pathways, such as 12-HHT, TXB_2_, and 12-HETE, molecules often overexpressed in various inflammatory diseases. In the study by Tejedor-Calvo and colleagues, truffle extracts from various species were characterized and classified based on their antioxidant capacities. The analysis was conducted using both the FRAP and ORAC methods, which yielded comparable results. *Tuber magnatum* was identified as the species with the highest antioxidant activity, followed by *Tuber indicum* and *Tuber melanosporum*, whereas *Tuber gennadii* showed the lowest values when compared to Trolox, which was used as an antioxidant reference standard [2] (Table 3) However, the fact that extracts from these different truffle species have shown varying effects across different disease models—effects that are not always directly proportional to their antioxidant activity—highlights that ROS-scavenging capacity is not the sole beneficial property of truffles. This, combined with the fact that truffles are natural, edible, readily available, and relatively affordable, positions them as a more comprehensive therapeutic and adjuvant option compared to conventional antioxidant molecules typically used for similar purposes.

### 4.3. Hepatoprotective Properties

In recent years, scientific research has strengthened the hypothesis that truffles may exert a protective role on the liver mainly through antioxidant and anti-inflammatory mechanisms, although further research on clinical models is needed to confirm this evidence. First, the liver-protective action of the desert truffle (*Terfezia claveryi*), native to Baghdad, was reported in a study by Janakat and Nassar (2010), who analyzed different extracts obtained with various solvents and compared them with the liver damage induced by carbon tetrachloride (CCl_4_) in male Wistar albino rats [82]. The research aimed to clarify the influence of three extraction methods—with water, methanol, and petroleum ether—on the hepatoprotective activity of the fungus. During the experiment, rats were individually treated with the three types of extracts twice daily for three consecutive days, before intraperitoneal administration of a toxic mixture of CCl_4_ and olive oil (2 mL CCl_4_/kg body weight). After induction of liver damage, the animals received two additional doses of the extracts, one hour and four hours apart. The results showed that the aqueous extract of *Terfezia claveryi* exerted a marked protective effect against CCl_4_ toxicity [82]. In addition to the pioneering study by Janakat and Nassar, several studies in recent years have explored the hepatoprotective potential of desert truffles. In particular, extracts of *Terfezia claveryi* were evaluated for acrylamide toxicity in rats, showing a significant improvement in liver biochemical markers (ALT (Alanine AminoTransferase), AST (Aspartate AminoTransferase) and bilirubin) and an increase in antioxidant capacity, resulting in protection of liver histology [83]. Another study on *Terfezia boudieri* demonstrated that the administration of aqueous extracts exerts both a preventive and curative effect on paracetamol-induced liver and kidney damage, reducing oxidative stress and limiting histopathological alterations [84] (Table 4).

### 4.4. Antitumor and Anticarcinogenic Potential

The immunomodulatory and antineoplastic potential of truffles remains poorly explored. Like other fungi, these ascomycetes are rich in polysaccharides. Compounds such as lentinan, crestin, and schizophyllan, isolated from fungal species, have already demonstrated significant effects in modulating the immune system and suppressing tumor growth [85]. In this context, various polysaccharides have been extracted from both fermentative cultures of *Tuber melanosporum*, *Tuber indicum*, *Tuber sinense*, and *Tuber aestivum*, and from the fruiting bodies of *Tuber indicum*, *Tuber himalayense*, and *Tuber sinense*, using activated charcoal columns. These compounds have shown cytotoxic activity in vitro against several tumor cell lines, including hepatocellular carcinoma (HepG2), lung adenocarcinoma (A549), colon carcinoma (HCT-116), breast cancer (SK-BR-3), and promyelocytic leukemia (HL-60), and were found to consist primarily of D-mannose, D-glucose, and D-galactose [16]. The limited availability of data in the literature therefore reflects a lack of targeted investigations rather than an absence of relevant biological properties, making the topic worthy of further investigation.

More recent studies have confirmed the interest in the antitumor activity of truffles. Methanolic extracts of *Tuber aestivum* and *Tuber magnatum* showed marked cytotoxicity in vitro on cervical (HeLa), breast (MCF-7), and colon (HT-29) cancer cells, while the aqueous extract was particularly effective against MCF-7 cells [10]. A recent study demonstrated that aqueous extracts of Terfezia claveryi, rich in bioactive compounds identified via gas chromatography-mass spectrometry, can be effectively used for the green synthesis of silver nanoparticles. These nanoparticles exhibited notable biological activities, including antioxidant, antibacterial, and moderate anticancer effects. Cytotoxicity testing revealed that the extract showed toxic effects against hepatocellular carcinoma (HCAM) cells, indicating potential therapeutic applications [86]. Furthermore, hexane and ethyl acetate extracts of the same species exerted cytotoxic effects on several tumor lines, including that of human glioblastoma (U-87 MG), with an IC_50_ of 50.3 µg/mL. It has been demonstrated that *Terfezia* and *Tuber gennadii* extracts exhibited capacity in inhibiting the cellular growth of several cancer cell lines, including NCI-H460, HeLa, HepG2, and MCF-7 cell lines, indicating anti-proliferative activity [87]. In addition to an antiangiogenic effect (aortic ring test), these extracts induced mitochondrial depolarization and nuclear condensation in treated cells. The observed activities were attributed to the presence of sterols and triterpenes such as stigmasterol, β-sitosterol, squalene, and lupeol [88]. In particular, phytosterols have been shown to inhibit cell proliferation and angiogenesis and stimulate apoptosis [89]. Stigmasterol, specifically, reduced lipid peroxidation, increased the activity of antioxidant enzymes (glutathione, superoxide dismutase, and catalase), and prolonged survival in mouse models of Ehrlich’s ascites carcinoma. Squalene, on the other hand, showed selective antiproliferative activity without interfering with major physiological processes.

Most truffle polysaccharides belong to the class of β-glucans, characterized by β-(1→3) chains with β-(1→6) branches. These polymers, and their derivatives, play a key role in biological processes such as cell communication, molecular recognition, and immune defense. Experimental evidence has shown that they can inhibit tumor development and dissemination by enhancing immune activity and stimulating lymphocytes, macrophages, and the production of cytokines (interferons, interleukins, and immunoglobulins) directed against neoplastic cells. For example, polysaccharides isolated from *Terfezia claveryi* caused cell cycle arrest in the G2 phase and a reduction in the G0/G1 population in Ehrlich ascites carcinoma cells [90]. Within the same class of compounds, fermentation fractions containing triple-stranded β-D-glucans have been shown to exert a more pronounced cytotoxic effect than those of fruiting bodies, while low-molecular-weight heteropolysaccharides are more active overall [16].

Lipids also contribute to the antitumor potential of truffles. Oleic acid, abundantly present, has been associated with the suppression of HER2 oncogene overexpression, increased intracellular production of ROS, and activation of caspase-3, thus promoting tumor cell apoptosis.

Finally, *Tuber melanosporum* fruiting bodies have been found to contain key enzymes of endocannabinoid metabolism, including NAPE-PLD, FAAH, DAGL, and MAGL, as well as anandamide, a lipid derivative with antineoplastic properties [29]. Anandamide has been observed to inhibit angiogenesis in highly invasive breast cells, induce apoptosis in colorectal carcinoma cells via COX-2-derived prostaglandin metabolites [91], and reduce tumor cell proliferation (Table 5).

### 4.5. Future Translational Perspectives

Although most studies on truffle bioactivity are still limited to in vitro assays, increasing evidence from preclinical models supports their therapeutic potential. Despite these promising preclinical outcomes, clinical trials in humans remain absent. To date, only a few studies have explored truffle-derived compounds in food or nutraceutical formulations, mainly focusing on safety and palatability rather than therapeutic endpoints. This highlights the need for controlled clinical investigations to confirm efficacy, bioavailability, and mechanisms of action in humans.

## 5. Extraction and Preservation Techniques for Bioactive Compounds

### 5.1. Extraction Methods

Conventional extraction methods for bioactive compounds in truffles include solvent extraction (using water, ethanol, or methanol), Soxhlet extraction, and distillation, but these often have limitations such as low efficiency, long extraction times [4]. Moreover, they may degrade thermo-sensitive compounds (e.g., volatiles, phenolics, anandamide) due to high temperatures or prolonged solvent exposure [92]. Despite these drawbacks, conventional methods are still used in early-stage studies to establish baseline chemical profiles, particularly for phenolics, sterols, and volatile analysis. Innovative techniques like ultrasound-assisted extraction (UAE), pressurized liquid extraction (PLE), supercritical CO_2_ extraction and microwave-assisted extraction (MAE), have been shown to significantly improve extraction yields, reduce solvent use, and better preserve the bioactivity of polysaccharides, phenolics, sterols, and volatiles [24,38,93,94].

Table 6 provides a summary of the extraction methods used to obtain bioactive compounds from truffles.

#### 5.1.1. Polysaccharides

Extraction with hot water or ethanol are the conventional and standard methods for extracting polysaccharides from truffles. These techniques involve relatively low extraction efficiencies and may not fully preserve bioactivity [38]. However, recent studies on species such as *Tuber huidongense* have optimized time and temperature using response surface methodology to maximize yield and antioxidant activity [69].

Innovative methods often reduce extraction times, significantly improving yield and bioactivity. For example, UAE increases the release of polysaccharides by breaking down cell walls and improving mass transfer. Studies report comparable or higher yields than HWE but with reduced time/energy requirements [95]. PLE and the use of subcritical water allow extraction under controlled temperature/pressure conditions that promote the release of β-glucans and chitins without the use of organic solvents. Recent applications on different truffle species have shown fractions rich in (1→3), (1→6)-β-D-glucans and other heteropolysaccharides [96]. Furthermore, the use of hydrolytic enzymes (cellulase, pectinase) before or during extraction helps to release wall-bound polysaccharides or polymer matrix, improving yield and sometimes selectivity towards fractions with high biological activity [97]. Studies have also reported improved yield following the coupling of extraction techniques. For example, MAE + UAE or enzymatic + UAE combinations have been explored to increase yield and reduce thermal degradation, with positive results on some truffle species [98]. Also combining natural deep eutectic solvents with UAE increased polysaccharide yield up to 11.5 times compared to conventional solvents, i.e., 1,3-butanediol extraction, while also enhancing antioxidant and skin-penetration properties [93]. PLE with optimal conditions (16.7 MPa, 180 °C, 30 min) yields 64% extract with water and 22.5% with ethanol, containing 9.1% β-glucan and 4.5% ergosterol [38]. UAE further improves efficiency, with yields ranging from 7.2% (*T. huidongense*, 99.65 W, 40.4 min, 70.1 °C) to 68.9% (*T. aestivum*, 75:1 liquid-solid ratio, 15 min, pH 6.5, 25% amplitude) depending on optimization and species [69,95]. Moreover, enzymatic extraction (using papain, trypsin, and pectinase) can increase the yield up to 46.9% of total polysaccharide in *Tuber aestivum* under optimal conditions (50 °C, pH 6.0, 90 min) [97]. Overall, innovative extraction methods like UAE, enzymatic extraction, and PLE significantly outperform conventional techniques in both yield and preservation of bioactivity.

#### 5.1.2. Phenolic Compounds

Traditional extraction with hydroalcoholic solvents (methanol/ethanol-water 50–80% *v*/*v*) or pure methanol is commonly used, but can result in low yields and a partial loss of bioactivity. Characterization of the phenolic profile of black summer truffle (*Tuber aestivum*) and white truffle (*Tuber magnatum*) also show different dominant phenolic compounds: p-hydroxybenzoic acid, baicalein, and kaempferol are most abundant in *Tuber aestivum*, while epicatechin and catechin dominate in *Tuber magnatum* [10]. However, to maximize yield and quality, advanced techniques can also be applied in combination with traditional methods. PLE applied to truffles has yielded phenolic fractions with measurable content and good antioxidant activity [99]. Studies applied to truffles indicate that UAE is effective in increasing yield and speed of phenolic compound extraction [100]. However, it was found that these techniques can alter the content and profile of phenolics, sometimes reducing total phenolic content, but modifying their biofunctional properties, such as antioxidant and enzyme inhibition activities [24]. UAE can alter the phenolic profile and sometimes reduce total phenolic content, but it may enhance specific bioactivities such as enzyme inhibition or antioxidant capacity in certain protein fractions, as seen in Tirmania nivea, where non-sonicated albumin fractions at 30 °C yielded the highest TPC (3.5–34.1 mg/g) and antioxidant activity (up to 91.9%) [24]. Enzyme treatment can release cell wall-bound phenols; when combined with UAE or MAE, this pre-treatment improves yield and selectivity in many plant and fungal matrices, and can also be applied to truffles to increase the recovery of bound phenolic compounds [101]. Thus, the extraction technique and procedures employed significantly affect both the yield and bioactivity of phenols.

#### 5.1.3. Volatile and Aroma Compounds

Conventional methods like steam distillation and Soxhlet extraction are less effective for preserving the complex aroma profile of truffles, often extracting a narrower range of volatiles and potentially degrading sensitive compounds [4,102]. However, some techniques allow the extraction of volatile compounds without the creation of artifacts or modification of the compounds. These are Headspace Solid-Phase Microextraction (HS-SPME), Stir-Bar Sorptive Extraction (SBSE) and Supercritical Fluid Extraction (SFE). HS-SPME is today the reference method for comparative profiling, as it is non-destructive, requires a small amount of sample, does not use solvents and is easily standardized. HS-SPME coupled with gas chromatography allows the identification of even hundreds of compounds depending on the analytical conditions (fiber polarity, temperature and extraction time, use of NaCl) [48,103,104]. SBSE offers superior adsorption capacity for low-abundance compounds and can reveal trace components that are not always captured by SPME in a short time. It is particularly useful when you want to conduct an extensive investigation of the less abundant fraction of the compounds responsible for the aroma, even if it requires longer exposure/extraction times [105]. SFE enables a richer (about 70% of yield) and more diverse array of volatile compounds, including acids, esters, and alcohols, and better preserving the natural aroma and flavor profile [4,94,102]. However, this technique is mainly used in industrial and experimental contexts [4].

#### 5.1.4. Sterols and Lipids

The Folch (chloroform:methanol 2:1 *v*/*v*) and Bligh & Dyer (two-step method with methanol/chloroform/water) methods remain the standard for total lipid extraction from biological matrices and are also widely used in truffle studies. They allow separation of the organic phase containing total lipids from the aqueous phase; from the organic fraction, saponification and separation of the unsaponifiable for sterol analysis are then performed. However, these methods involve the use of solvents and possible yield variations related to sample composition [106,107]. Innovative techniques do not involve the use of solvents and an adequate yield. PLE with water and ethanol yields extracts containing 9.1% β-glucan and 4.5% ergosterol, with ethanol extracts also containing fatty acids and fungal sterols such as brassicasterol and stigmasterol [38,96]. Supercritical CO_2_ extraction under optimal conditions (2.27 mg/mL CO_2_ flow, 82.5 min, 40 °C, 30 MPa) efficiently extracts sterols (e.g., brassicasterol, ergosterolo) and fatty acids (oleic and linoleic acid) in similar amounts across truffle species [94]. The lipid and sterol yield of truffles is generally low and varies according to species, maturity, sample preparation and extraction technique [19,23]. However, studies show the diagnostic potential of the lipid class in the commercial tracking of high-value species (*T. magnatum*, *T. melanosporum*) [108].

**Table 6:** Summary of extraction techniques used for bioactive compounds from truffles. The table reports conventional and advanced methods, highlighting the benefits of using the methods.

Compound Class	Conventional Methods	Advanced Methods	Benefits of Advanced Methods
Polysaccharides	Hot water or ethanol [36]	UAE [93] PLE [94] Enzymatic (Cellulase, Pectinase) [95] MAE + UAE [96] Enzymatic + UAE [96] NADES + UAE [91]	UAE and PLE reduce processing time, increase yield and bioactivity. PLE produces β-glucans and chitosans. Enzymes release bound polysaccharides. NADES + UAE increase yield up to 11.5 times.
Phenolic Compounds	Hydroalcoholic solvents or pure methanol [8]	PLE [97] UAE [98] Enzymatic + UAE or MAE [99]	Increased yield and reduced processing times Can reduce total phenolic content but improve specific bioactivities.
Volatile and Aroma Compounds	Steam distillation [4,100] Soxhlet extraction [100]	HS-SPME [46,101,102] SBSE [103] SFE [4,92,100]	HS-SPME does not alter the aroma profile. SBSE has a higher adsorption capacity for trace compounds. SFE produces a more diversified aroma profile.
Sterols and Lipids	Folch [106] Bligh & Dyer [107]	PLE [36,94] Supercritical CO_2_ [92]	PLE produces beta-glucan and ergosterol. Supercritical CO_2_: extracts sterols and fatty acids efficiently and without organic solvents

### 5.2. Preservation Techniques to Maintain Bioactivity

The short shelf life and high sensitivity of many of these molecules to oxidation, hydrolysis, enzymatic degradation and microbial activity make it crucial to develop preservation strategies that maintain both their chemical profile and bioactivity. In order for the chemical characteristics and bioactivity to be preserved for a long time, it is important to take action immediately after harvesting. In fact, storage at low temperatures (4 °C) slows down enzymatic activity and microbial growth. Freezing (−20 °C) further reduces activity, but can alter tissue structure, promoting subsequent changes in VOCs upon defrosting [109,110].

Lyophilisation is often referred to as the technique that best preserves the compositional characteristics of products, because it removes water at low temperatures, limiting thermal reactions. However, depending on the parameters used (time, temperature, atmosphere), this technique can also result in quantitative modification of some of the more volatile VOCs and alter antioxidant activity. The combined use of lyophilisation with appropriate protectors (e.g., cryoprotectants, antioxidants) has been proposed to mitigate losses [111]. The use of modified atmosphere packaging (MAP), active O_2_/CO_2_ control and gas barrier packaging can slow down respiration, microbial growth and oxidation of sensitive compounds, helping to preserve volatile flavor components and microbial balance. Studies on *Tuber melanosporum* and *Tuber aestivum* show an extension of shelf life and maintenance of the sensory profile [51]. However, applying drastic conditions (e.g., very low O_2_) can cause a modification of the aroma [112].

To maintain the shelf-life of truffles and the bioaccessibility of polar and lipophilic compounds, micro- and nano-encapsulation techniques can be employed [113,114]. In black Périgord truffles (*Tuber melanosporum*), encapsulation using β-cyclodextrin or γ-cyclodextrin has been shown to stabilize key volatile organic compounds (VOCs) [115,116]. For example, the paste method of β-cyclodextrin encapsulation retained truffle volatiles more effectively than direct mixing, and by day 90, encapsulated truffles showed less volatile loss than freeze-dried samples [115]. Similarly, γ-cyclodextrin was able to encapsulate and preserve 30 different truffle VOCs, including important aroma markers, with minimal changes during storage [116]. Furthermore, the combination of lyophilisation and encapsulation allows for a longer shelf life and better preservation of functional properties than either technique alone [115].

The use of edible coatings has also proven effective in reducing volatile loss and maintaining truffle quality, while the use of antioxidants or antimicrobial agents alone has been less successful [116]. Edible coatings, such as chitosan, gum Arabic, and gelatin hydrogels, can delay changes in aroma, reduce microbial spoilage, and help preserve the sensory and biochemical qualities of truffles during refrigerated storage [117,118].

Gamma irradiation at appropriate doses (up to 2.5 kGy) can significantly reduce microbial loads and extend shelf life without negatively affecting most bioactive compounds or sensory properties, though some minor losses in sterols may occur and phenolic content may even increase during storage [119,120]. Studies on the application of High-Pressure Processing (HPP) to truffle-based products have shown that the products retain an aroma profile closer to freshness than other thermal stabilization methods, although pressure/time tuning is required [121].

Additionally, the use of natural antimicrobials like gallic acid can control spoilage bacteria and preserve truffle quality without introducing off-flavors [110]. These strategies, especially when combined, are effective in maintaining the bioactivity and unique qualities of truffles for food and ingredient applications. These extraction and preservation strategies enable the application of truffle bioactive compounds in the food industry (as natural flavorings and functional ingredients), nutraceuticals (for antioxidant, anti-inflammatory, and antidiabetic effects), and pharmaceuticals (for cytotoxic, immunomodulatory, and enzyme inhibitory activities) [38,92,96,115,122].

## 6. Truffles as a Functional Food

As previously discussed, truffles are a rich natural source of bioactive compounds with significant antioxidant properties, including phenolics, flavonoids, sterols, carotenoids, and various vitamins [71,123]. Their popularity is generally attributed not only to their nutritional value but also to their distinctive and sophisticated aroma and taste. Wild edible fungi, such as truffles, are widely recognized not only for enhancing the flavor of otherwise bland staple foods but also for their intrinsic nutritional and health-promoting properties. Historically celebrated as “underground gold” and “the diamond of the kitchen” [92], truffles combine cultural prestige with scientific relevance, reinforcing their role as both a luxury food and a potential functional ingredient. The antioxidant molecules they contain are involved in neutralizing reactive oxygen species (ROS) and mitigating oxidative stress through various mechanisms, including electron donation, hydrogen atom transfer, and singlet oxygen quenching [123,124]. Truffles exhibit a favorable nutritional composition, with low lipid content but a high proportion of unsaturated fatty acids, proteins, and carbohydrates, making them a balanced food with promising antioxidant functionality [123,125]. Beyond their nutritional value, multiple studies have emphasized the broader biological activities of truffles, including anti-inflammatory, immunomodulatory, antimutagenic, anticarcinogenic, antimicrobial, and hepatoprotective effects [53,71,126,127,128]. These functional properties reinforce the notion that truffles may serve not only as gourmet delicacies but also as potential sources of bioactive compounds with therapeutic applications.

### 6.1. Perspectives in the Prevention of Chronic Diseases

Oxidative stress is widely recognized as a key etiological factor in the development of several chronic non-communicable diseases, including cardiovascular disorders, cancer, diabetes, and neurodegenerative conditions [129,130]. Accordingly, the regular consumption of natural antioxidants has been proposed as a promising strategy to mitigate the onset and progression of these diseases. Truffles, owing to their richness in bioactive compounds, may contribute meaningfully to this preventive approach [123,131]. Specific constituents isolated from truffles, such as polysaccharides, sterols (e.g., tuberoside), and unsaturated fatty acids, have demonstrated anti-inflammatory, immunomodulatory, and antimutagenic activities. Furthermore, these compounds are known to reduce lipid peroxidation and modulate cytokine production [127]. Extracts derived from *Tuber* species, particularly methanolic fractions, have shown notable free radical scavenging capacity and elevated polyphenolic content, reinforcing their potential to protect against oxidative damage [123]. Taken together, these results highlight the potential role of truffles as functional foods and natural sources of therapeutic agents with applications in the prevention of chronic non-communicable diseases [125,132].

### 6.2. Applications in Functional Foods and Supplements

Traditionally, truffles have been consumed fresh or incorporated into gourmet products such as truffle oils, butters, cheeses, and sauces, where they serve as both flavor enhancers and nutritional enrichers [133]. Recently, interest has shifted toward standardized extracts enriched in phenolics, flavonoids, sterols, and polysaccharides for use in functional beverages, dairy products, and dietary supplements, aiming to combine sensory appeal with evidence-based health benefits [125,134]. Desert truffles (*Terfezia*, *Tirmania*) and Asian species, due to their lower cost, represent promising alternatives for the development of functional formulations [128].

A critical challenge for large-scale use lies in their short postharvest shelf-life [135]. Traditional storage methods such as dry wrapping or immersion in rice are still used but present significant limitations, including aroma depletion and microbial deterioration [3,136]. Modern strategies, including refrigeration, vacuum packaging, and modified atmosphere packaging (MAP), have proven more effective in preserving aroma and texture for up to 2–4 weeks [45,112]. Emerging approaches such as irradiation, ultrasound-assisted processing, and active packaging with antimicrobial biopolymers like chitosan have shown promising results in extending shelf-life without compromising nutritional or sensory properties [3].

These preservation strategies are essential not only for maintaining the culinary and sensory qualities of truffles but also for enabling their successful integration into the growing markets of functional foods and nutraceuticals.

### 6.3. Other Applications and Future Perspectives

In addition to their culinary applications, truffles have also gained interest in the cosmetic, fragrance, and pharmaceutical industries, where bioactive extracts are valued for their antioxidant, anti-aging, and skin-enhancing properties [3]. Furthermore, due to their unique metabolic features associated with mycorrhizal symbiosis, truffles serve as useful biological models for investigating enzymatic adaptations to low-oxygen (microaerobic) environments.

From a broader perspective, the increasing global demand for truffles, especially within European markets, underscores the need for sustainable cultivation practices and advanced preservation technologies. The integration of traditional ecological knowledge with modern biotechnological tools holds promise for accelerating the development of truffle-based functional products, potentially enabling a shift from exclusive gourmet use to more accessible nutraceutical and therapeutic applications.

## 7. Conclusions and Prospects

The gastronomic value of truffles, linked to their distinctive aroma and flavor, has fueled strong global demand. However, their appeal is not limited to their culinary value, as these hypogeal fungi also represent a valuable source of nutrients and bioactive molecules, with no significant adverse effects reported. Several Tuber species display a rich nutritional profile, including carbohydrates, proteins, dietary fiber, essential minerals, fatty acids, and amino acids. Beyond their nutritional composition, there is growing interest in their therapeutic potential. Recent studies have highlighted the presence of functional compounds such as phenols, flavonoids, and polysaccharides, which are attributed with antioxidant, antimicrobial, anti-inflammatory, antidiabetic, and even antitumor properties. However, despite growing interest in their biochemical composition and biological effects, research in this field is still in its infancy. Most studies have been limited to in vitro assays or preclinical models, while clinical trials and in-depth in vivo studies remain scarce. This represents a significant gap that must be filled to validate the promising health benefits attributed to truffles and to better elucidate their mechanisms of action. Furthermore, considerable variability in truffle bioactivity has been reported, largely influenced by species, geographic origin, stage of ripeness, and extraction methods. Standardization of analytical procedures and extraction techniques is therefore essential to ensure reproducibility and comparability between studies. Another critical challenge concerns the stability of bioactive compounds during processing and storage, which must be optimized to preserve their functional properties. From an application perspective, the diverse spectrum of truffle metabolites, including polysaccharides, sterols, fatty acids, and unique molecules such as anandamide, positions these mushrooms as promising candidates for the development of nutraceutical formulations and functional foods. Furthermore, their potential in pharmaceutical applications, particularly in oncology and immunomodulation, deserves further exploration. Advances in nanotechnology, encapsulation systems, and novel drug delivery strategies could further improve the bioavailability and efficacy of truffle-derived compounds. Therefore, future research should focus on improving extraction techniques, aiming to maximize the yield of bioactive compounds present in truffles. At the same time, it will be crucial to expand biological testing on various diseases and microorganisms, thus concretely enhancing the therapeutic and functional potential of these metabolites in natural products and pharmaceutical applications. In this context, the adoption of innovative technological strategies could help overcome the critical issues related to truffle preservation, extending their shelf life without altering their chemical profile or compromising their safety. These developments would pave the way for a more widespread and targeted use of truffles in both nutraceutical and medicinal fields.

In conclusion, truffles should no longer be considered exclusively as luxury culinary delicacies, but as emerging reserves of natural antioxidants and bioactive molecules. Filling current research gaps with well-designed in vivo studies and clinical trials will be crucial to unlocking their full potential and promoting their integration into preventive and therapeutic approaches for human health.

## Figures and Tables

**Table 1:** Summary of the main nutritional and bioactive components of selected truffle species. The table highlights differences in macronutrient content, predominant fatty acids, and key functional compounds, including phenolics, flavonoids, and sterols.

Species	Main Carbohydrates (% DW)	Protein (% DW)	Fat (% DW)	Key Fatty Acids	Notable Bioactives	Main Minerals *	Origin (Region/Country)	References
*Terfezia claveryi*	60–80	12–18	1–4	Linoleic, (~35%), Oleic (~30%), Palmitic (~20%) → PUFA > MUFA	Rutin, gallic acid, flavonoids, carotenoids, polyphenols, essential amino acids	K, Na, Fe, Cu, Zn, Ca, P, Mg, Mn, Ni	Saudi Arabia	[5,6,7,12]
*Terfezia boudieri*	55–68	12–28	3–5	Linoleic (up to 54%)	Polyphenols, flavonoids, terpenoids, phytosterols, anthocyanins, ascorbic acid	K, P, Fe, Ca, Na, Mg, Zn, Cu	Turkey/Tunisia/Saudi Arabia	[12,13,14]
*Tirmania nivea*	50–65	12–27	2–7	Linoleic, Oleic	Polyphenols, flavonoids, carotenoids, vitamin C	K, P, Fe, Ca, Na, Mg, Zn, Cu, Mn	Saudi Arabia/Tunisia	[5,6,13,15]
*Tuber aestivum*	60–79	14–24	1–5	Oleic (40–50%), linoleic (20–30%), palmitic (10–15%)	Polyphenols, flavonoids, sterols	K, P, Fe, Ca, Mg, Zn, Cu	Italy/France	[2,13]
*Tuber melanosporum*	74–75	12–18	2–4	Oleic (~45%), linoleic (~25%), palmitic (~15%) → MUFA > PUFA	Ergosterol, phenolics	K, P, Fe, Ca, Mg, Zn, Cu	France/Spain	[13]
*Tuber magnatum*	60–75	10–22	1–5	Palmitic > oleic > linoleic	High phenolics, ergosterol	K, P, Fe, Ca, Mg, Zn, Cu	Italy	[2,13]
*Tuber indicum*	52–80	13–23	2–7	Linoleic (~40–45%), Oleic (~25–30%), Palmitic (~15%) → PUFA > MUFA	Phenolics, tocopherols, ergosterol	K, P, Ca, Mg, Fe, Zn	China	[2,11]
*Tuber sinense*	65–70	14–20	3–4	Oleic (~40%), Linoleic (~30%), Palmitic (~15%) → MUFA ≈ PUFA		K, P, Ca, Mg, Fe, Zn	China	[16]
*Picoa juniperi*	37	22.5	20	Linoleic, Oleic	Polyphenols, fiber	K, P, Fe, Ca, Mg, Zn, Cu	Turkey/Spain	[6]

* Main minerals: Potassium (K) and phosphorus (P) are generally the most abundant, followed by iron (Fe), calcium (Ca), sodium (Na), magnesium (Mg), zinc (Zn), copper (Cu), manganese (Mn), and nickel (Ni).

**Table 2:** Summary of the antiviral, antibacterial, and antimicrobial effects of different types of truffles.

Truffles	Antiviral, Antibacterial and Antimicrobial Effects	References
*Terfezia claveryi*	• 40% inhibition of the growth of *Pseudomonas aeruginosa* • 66.4% inhibition of the growth of *Staphylococcus aureus*	[53,54]
*Tuber nivea*	• 70% al 100% inhibition of the growth of *Pseudomonas aeruginosa* and *Staphylococcus aureus* • antimicrobial activity against three Gram-positive and four Gram-negative bacteria, including *Salmonella typhimurium*, *Escherichia coli*, *Pseudomonas aeruginosa*, *Enterococcus faecalis*, *Staphylococcus aureus*, *Staphylococcus epidermidis*, and *Bacillus subtilis*	[54,56]
*Terfezia boudieri*	Antimicrobial activity against both Gram-positive and Gram-negative bacteri	[55]
*Tirmania* sp.	Antibacterial effect (Gram-positive and Gram-negative)	[56]
*Tuber pinoyi*	Limit the proliferation of *Staphylococcus aureus*	[58,59]

**Table 3:** Summary of the antioxidant and anti-inflammatory activities of different types of truffles.

Truffles	Antioxidant and Anti-Inflammatory Activities	References
*Terfezia boudieri*	Scavenging activity against DPPH (2,2-diphenyl-1-picrylhydrazyl) radicals	[55]
*Tuber huidongense*	High radical-scavenging capacity	[68,69]
*Tuber leonis*	High radical-scavenging capacity	[68,69]
*Tuber pinoyi*	High radical-scavenging capacity	[68]
*Terfezia claveryi*	• high activity in inhibiting lipid peroxidation • inhibits oxidative damage at liver level	[82]
*Picoa juniperi*	High activity in inhibiting lipid peroxidation	[71]
*Tuber nivea*	• high DPPH radical scavenging capacity • greater efficacy against NO radicals • high level of inhibition of deoxyribose degradation	[73]
*Tuber melanosporum*	Activation of the Nrf2 and NF-κB pathways and the enhancement of antioxidant defenses, both enzymatic (superoxide dismutase, catalase) and non-enzymatic (vitamins C and E)	[2]
*Tuber magnatum*	Inhibits pro-inflammatory metabolites derived from the COX-1 and 12-LOX pathways, such as 12-HHT, TXB_2_, and 12-HETE	[2,10]

**Table 4:** Summary of the hepatoprotective properties of truffles.

Truffles	Antioxidant and Anti-Inflammatory Activities	References
*Terfezia claveryi*	• exerts a marked protective effect against CCl_4_ toxicity • improves liver biochemical markers (ALT (Alanine AminoTransferase), AST (Aspartate AminoTransferase) and bilirubin) and increases antioxidant capacity, resulting in protection of liver histology	[82,83,84]

**Table 5:** Summary of the antitumor and anticarcinogenic potential of different types of truffles.

Truffles	Antitumor and Anticarcinogenic Potential	References
*Tuber aestivum*	Marked cytotoxicity in vitro on cervical (HeLa), breast (MCF-7), and colon (HT-29) cancer cells	[10,16]
*Tuber magnatum*	Marked cytotoxicity in vitro on cervical (HeLa), breast (MCF-7), and colon (HT-29) cancer cells	[10]
*Terfezia claveryi*	• toxic effects against hepatocellular carcinoma (HCAM) cells • inhibiting the cellular growth of several cancer cell lines, including NCI-H460, HeLa, HepG2, and MCF-7 cell lines • causes cell cycle arrest in G2 phase and a reduction in the G0/G1 population in Ehrlich ascites carcinoma cells	[87,90]
*Tuber gennadii*	Inhibiting the cellular growth of several cancer cell lines, including NCI-H460, HeLa, HepG2, and MCF-7 cell lines	[87]
*Tuber melanosporum*	Induce apoptosis in colorectal carcinoma cells via COX-2-derived prostaglandin metabolites, and reduce tumor cell proliferation	[29,91]

## Data Availability

No new data were created or analyzed in this study. Data sharing is not applicable to this article.

## Abbreviations

The following abbreviations are used in this manuscript:

2-AG	2-Arachidonoylglycerol
12-HETE	12-Hydroxyeicosatetraenoic acid
12-HHT	12-Hydroxyheptadecatrienoic acid
BMTM	bis(methylthio)methane
CLs	Cardiolipins
COX-1	Cyclooxygenase-1
DAGL	Diacylglycerol Lipase
DPPH	2,2-Diphenyl-1-picrylhydrazyl (antioxidant radical-scavenging assay)
DW	Dry weight
EC_50_	Effective Concentration 50%
FAAH	Fatty Acid Amide Hydrolase
FRAP	Ferric Reducing Antioxidant Power (antioxidant assay)
GAE	Gallic Acid Equivalents (reference for phenolic content)
GC–MS	Gas Chromatography–Mass Spectrometry
GC–O	Gas Chromatography–Olfactometry
HPP	High-Pressure Processing
HS-SPME	Headspace Solid-Phase Microextraction
HWE	Hot Water Extraction
ICP–MS	Inductively Coupled Plasma–Mass Spectrometry
IC_50_	Inhibitory Concentration 50%
IMP	Inosine-5′-monophosphate
LC–MS	Liquid Chromatography–Mass Spectrometry
LOX (12-LOX)	12-Lipoxygenase
LPCs	Lysophosphatidylcholines
MAE	Microwave-Assisted Extraction
MAGL	Monoacylglycerol Lipase
MAP	Modified Atmosphere Packaging
MPa	Megapascal
MUFA	Monounsaturated Fatty Acids
NAPE–PLD	N-Acyl-Phosphatidylethanolamine-Phospholipase D
NO	Nitric Oxide
OAV	Odor Activity Value
ORAC	Oxygen Radical Absorbance Capacity (antioxidant assay)
P&T–GC–MS	Purge-and-Trap Gas Chromatography–Mass Spectrometry
PAs	Phosphatidic Acids
PLE	Pressurized Liquid Extraction
PUFA	Polyunsaturated Fatty Acids
ROS	Reactive Oxygen Species
SBSE	Stir-Bar Sorptive Extraction
SFA	Saturated Fatty Acids
SFE	Supercritical Fluid Extraction
TGs	Triacylglycerols
TPC	Total Phenolic Content
TXB_2_	Thromboxane B_2_
UAE	Ultrasound-Assisted Extraction
UHPLC–QE	Ultra-High-Performance Liquid Chromatography coupled with Q Exactive
Orbitrap–MS	Orbitrap Mass Spectrometry
UV	Ultraviolet
VOCs	Volatile Organic Compounds
